# Survival of the Synesthesia Gene: Why Do People Hear Colors and Taste Words?

**DOI:** 10.1371/journal.pbio.1001205

**Published:** 2011-11-22

**Authors:** David Brang, V. S. Ramachandran

**Affiliations:** Department of Psychology, University of California, San Diego, La Jolla, California, United States of America

## Abstract

Synesthesia is a perceptual experience in which stimuli presented through one modality will spontaneously evoke sensations in an unrelated modality. The condition occurs from increased communication between sensory regions and is involuntary, automatic, and stable over time. While synesthesia can occur in response to drugs, sensory deprivation, or brain damage, research has largely focused on heritable variants comprising roughly 4% of the general population. Genetic research on synesthesia suggests the phenomenon is heterogeneous and polygenetic, yet it remains unclear whether synesthesia ever provided a selective advantage or is merely a byproduct of some other useful selected trait. Progress in uncovering the genetic basis of synesthesia will help us understand why synesthesia has been conserved in the population.

## Abstract 2

This Unsolved Mystery reviews the biological evidence for why synesthesia, a condition in which stimuli presented through one modality spontaneously evoke sensations in an unrelated modality, may have been conserved in the population.

## Introduction

Synesthesia is a condition present in 2%–4% of the population [1] in which a sensory stimulus presented to one modality elicits concurrent sensations in additional modalities [2]. Synesthesia can theoretically bind any two senses, but research has largely focused on two of the most common variants in which auditory tones and achromatic (colorless) numbers produce vivid and perceptually salient colors. The specificity of these evoked colors remains stable over time within any given individual [3], but the same tone or grapheme doesn't necessarily evoke the same color in different people. Synesthesia has been of interest to scientists for nearly 200 years [4], and while familial linkage analyses show a strong genetic component, the precise genes involved and reasons why synesthesia has been conserved in the population remain unsolved mysteries.

## Neural Basis of Synesthesia

The neural substrate of synesthesia has been thoroughly studied in grapheme-color synesthesia (in which numbers and letters evoke colors) using both psychophysical tests and functional imaging. Several groups have demonstrated that simple achromatic graphemes activate both grapheme regions as well as color area V4 (a region of visual cortex that shows a stronger response to colors than to grayscale stimuli) in the brains of synesthetes, which is consistent with the view that synesthetic colors are sensory in nature (i.e., arise through a bottom-up processing stream) [5],[6] as opposed to being high-level cognitive associations, as has been proposed [7]. Predicting this finding of “cross-activation” between grapheme and color regions, Ramachandran and Hubbard [6] proposed that synesthesia results from an excess of neural connections between associated modalities, possibly due to decreased neural pruning between (typically adjacent) regions that are interconnected in the fetus. Consistent with this suggestion, a number of studies have demonstrated anatomical differences in the inferior temporal lobe near regions related to grapheme and color processing in synesthetes, including increased fractional anisotropy (reflecting increased white matter or coherence of white matter) [8],[9] and increased gray matter volume [9],[10]; increased connectivity has been found in other forms of synesthesia as well [11]. Furthermore, Brang and colleagues recently demonstrated that color area V4 becomes active as early as 110 ms after viewing achromatic letters and numbers, signifying that synesthetic colors follow a similar time-course in the brain as colors evoked from the retina [12]–[14].

**Figure 1:**
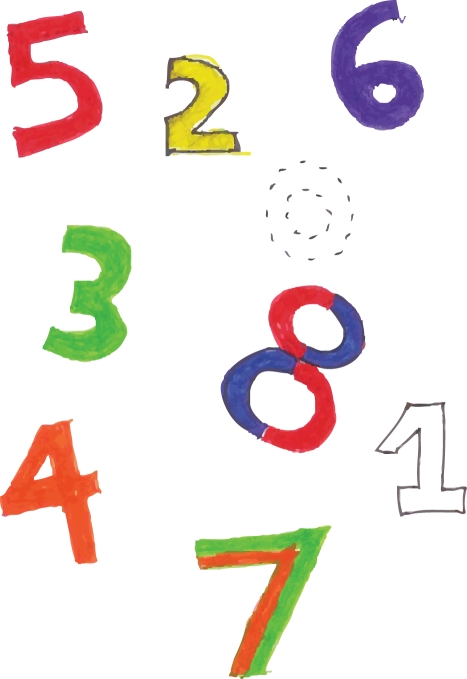
Number-color associations for one of our synesthetes.

Adding support to the sensory cross-activation hypothesis, Ramachandran and Hubbard [6],[15] demonstrated that synthetically induced colors can lead to perceptual texture segregation; recent results by Jamie Ward and colleagues [16] lend additional empirical evidence for this view. Ramachandran and Brang also noted [17] that the same number can take on multiple colors in some synesthetes (e.g., the numbers 7 and 8 in Figure 1) or even the visual appearance of textural qualities like metallic and smooth [18], further suggesting that synesthesia is a bottom-up sensory phenomenon. Nevertheless, top-down influences (e.g., attention, context, etc.) must also play a significant role in synesthesia as first shown by Ramachandran and Hubbard [15] using Navon figures; that is, if the subject sees a large 5 made up of small 2 s, the evoked color will change depending on whether the subject focuses on the local or global image (i.e., the “trees” or “forest”). How integration of these bottom-up and top-down processing streams occurs is still not known.

## Heredity in Synesthesia

**Figure 2:**
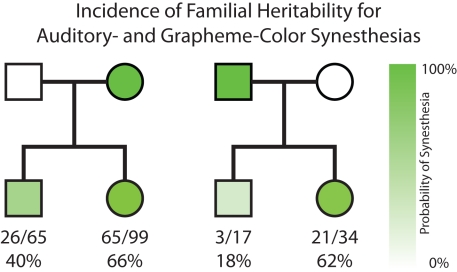
Average familial pedigrees for synesthetes composed from data in references [**3]**,[18],[19],[23],[25],[48****], demonstrating incidence of heritable transmission from one synesthetic parent (top row) to either a female or male child (bottom row). Squares represent males and circles females. Color intensity reflects probability of synesthesia pedigree taken from the numbers at the bottom, representing incidence of each case.

While a proven genetic basis for synesthesia remains elusive, the phenomenon tends to run in families, as ∼40% of synesthetes report a first-degree relative with the condition [3],[19]. Pedigree analyses of synesthesia suggest high transmissibility from parent to offspring (Figure 2), yet in at least one confirmed instance, synesthesia is present in only one monozygotic twin [20]. At least 60 different forms of synesthesia have been documented (i.e., different combinations among the senses), reflecting the extreme heterogeneity of the condition, and one could easily assume that each type of synesthesia is caused by a unique gene or set of genes. However, the specific form of synesthesia an individual expresses can vary within families [19], suggesting the genetic undertones impose a predisposition to synesthesia but not its expression. Indeed, individuals with one type of synesthesia are much more likely to have another as well, an observation that was adduced by Ramachandran and Hubbard [15] as support for the idea that the defective pruning gene or genes confer a general propensity to linking unrelated sensations or even concepts. Further, while individual synesthetes often display multiple forms of the phenomenon, large-scale factor analyses suggest that some variants co-occur with greater frequency within a single individual, suggesting that some forms are more highly related (i.e., local “clustering” or “islands” of synesthesia types), which is suggestive of a common origin [21]. However, non-clustering forms still co-occur with greater frequency than predicted by prevalence rates in the general population [22], significantly impeding theories of single genetic markers and the notion of independence among different forms of the condition.

Previous studies examining the prevalence of synesthesia found a significant gender gap with a 6∶1 ratio of female synesthetes to males, leading to the suggestion that synesthesia was an X-linked condition [23]. However, prevalence studies conducted using random sampling have shown an even distribution of synesthesia among the genders, suggesting the discrepancy was based on methodological flaws and self-report biases in earlier studies. Subsequent research on the genetics of synesthesia has unfortunately not clarified which genes underlie the phenomenon. Examining the relationship between synesthesia-like hallucinogenic experiences and serotonin 2A receptor coding on chromosome 13, Brang and Ramachandran [24] suggest that synesthesia might occur from over-expression of this gene, producing higher receptor density. However, direct investigations using a whole-genome linkage scan [25] and a family-based linkage analysis [26] each localized distinct loci that overlapped neither with the region suggested by Brang and Ramachandran [24] nor with one another, suggesting either a lack of power from the derived sample sizes or that the phenomenon is largely polygenic. A polygenic origin is likely, as transmissibility is heterogenous and synesthetes often possess multiple forms, as mentioned above [15],[19]. In light of these conflicting results, research into the genetics underlying synesthesia remains in a nascent state and will require much larger sample sizes and variants of the condition to understand the underlying factors for transmission. Furthermore, evidence suggests that synesthesia may in fact be a graded phenomenon in the general population, creating the possibility that non-synesthetic relatives of synesthetes show endophenotypes of the condition, complicating the use of family linkage analyses.

## Why Was the Gene Conserved?

### Epiphenomenal

Before asking why a synesthesia gene might have been preserved through evolution, one first must consider the possibility that synesthesia may likely be merely epiphenomenal, and that the gene(s) involved may have served some totally unrelated purpose. It is also possible that the gene(s) may have been retained simply because they did not incur a great enough cost to be purged by selection, and could be an example of an evolutionary spandrel. Another possible explanation is that synesthesia simply represents the tail end of a normal distribution of cross-modality interactions present in the general population [15],[23]. Partial evidence supporting this idea comes as sensory deprivation and deafferentation (i.e., loss of sensory input through the destruction of sensory nerve fibers) can lead to synesthetic-like experiences. For example, after early visual deprivation due to retinitis pigmentosa, touch stimuli can produce visual phosphenes [27], and after loss of tactile sensation from a thalamic lesion, sounds can elicit touch sensations [28]. More remarkably, arm amputees experience touch in the phantom limb merely by watching another person's hand being touched [29]. Long-standing evidence has also demonstrated that hallucinogenic drugs can cause synesthesia-like experiences (for a review see [30]), suggesting the neural mechanism is present in all or many individuals but is merely suppressed. However, no research has yet established the relationship between these acquired forms to the genetic variant and whether the same neural mechanism is responsible for both.

### Creativity and Metaphor

The question of whether the synesthesia gene(s) may have a “hidden agenda” like the sickle cell anemia gene has with malaria resistance, and whether that agenda may be “creativity and metaphor”, was first raised by Ramachandran and Hubbard [15]. Subjectively, synesthetes report these experiences are largely positive and engender facilitative benefits for creative aspects of their lives. Studies have indeed confirmed the increased incidence of synesthesia among artists [31] and, relative to controls, synesthetes report spending more time engaged in creative activities [32]. However, the nature of the link between synesthesia and creativity (including metaphor) remains elusive given that synesthesia involves arbitrarily connecting two unrelated things (e.g., color and number), whereas there is a non-arbitrary conceptual connection between, say, Juliet and the sun (of *Romeo and Juliet*). One potential solution to this problem comes from realizing that any given word has only a finite set of associations (e.g., the sun is warm, nurturing, radiant, bright, etc.). The overlapping region among halos of associations between two words (e.g., Juliet and the sun; both are radiant, warm, and nurturing)—the basis of metaphor—exists in all of us but is larger and stronger in synesthesia as a result of the cross-activation gene; in this formulation synesthesia is not synonymous with metaphor, but only that the gene which produces synesthesia confers a propensity towards metaphor. While the link between synesthesia and creativity has received remarkable interest over the last decade, research has not yet directly demonstrated any causal relationship between the two and so the argument, at this point, remains seductive and compelling but not conclusive.

### Sensory Processing and Cognitive Abilities

Recently, research has confirmed numerous cognitive and perceptual benefits that are associated with synesthesia, any of which could be argued to produce a stronger basis for selection. As extreme examples of these benefits, two well-characterized savants have demonstrated remarkable memory abilities based on their synesthesias: Daniel Tammet used his synesthesia to memorize pi to 22,514 digits and Luria [33] described an individual (“S”) with a prodigious memory based largely on using synesthetic associations evoked by the items to be memorized. Such cases have led to the suggestion that synesthesia may exist as a foundation for savantism, and while Tammet and S are at the extreme end of the spectrum, synesthetes as a group also have demonstrated improved memories relative to controls, particularly for items related to their synesthetic experiences (e.g., memory for phone numbers is aided by number-color associations) [6],[15],[34],[35].

Outside the realm of memory research there is accumulating evidence of enhanced sensory processing in synesthesia as well. Specifically, grapheme-color synesthetes show enhanced detection of colors on a perceptually low-level visual test of parvocellular processing (K. Wagner, D. Brang, V. Ramachandran, K. Dobkins, unpublished data) paralleled by the finding of differences in early visual processes to simple colors using visual-evoked potentials [36] well before the time period in the brain in which synesthesia engages. Furthermore, number-color synesthetes are also more sensitive at discriminating very similar colors [37]. These early perceptual effects suggest that synesthesia is associated with increased processing of color information; however, it remains possible that these differences in color processing are not due to synesthesia itself but are merely caused by synesthetes' excessive experience with colors. Confirming these sensory enhancements in a different form of the condition, Banissy and colleagues demonstrated that mirror-touch synesthetes (individuals who experience tactile sensations on their own body while watching someone else being touched) possess increased tactile acuity [37]. Lastly, individuals who experience auditory sounds evoked by visual motion process rhythmic visual stimuli more accurately than controls and in a manner similar to how non-synesthetes process auditory information [38].

These demonstrations of enhanced processing of sensory information suggest a provocative evolutionary hypothesis for synesthesia: synesthetic experiences may serve as cognitive and perceptual anchors to aid in the detection, processing, and retention of critical stimuli in the world; in terms of memory benefits, these links are akin to a method of loci association. In addition to facilitating processes in individual sensory modalities, synesthetes also show increased communication between the senses unrelated to their synesthetic experiences, suggesting that benefits from synesthesia generalize to other modalities as well, supporting their ability to process multisensory information [39]. Furthermore, others have argued that synesthesia is the direct result of enhanced communication between the senses as a logical outgrowth of the cross-modality interactions present in all individuals [40]. Taken collectively, these data suggest that synesthesia may be associated with enhanced primary sensory processing as well as the integration between the senses. However, to fully understand this relationship, identification of the genetic component involved and evidence of selection on these genes will be required to determine whether synesthesia serves to enhance normal sensory processing.

## Towards a Solution

Synesthesia is associated with a wide variety of conceptual and perceptual benefits, suggesting that the gene(s) involved may have been selected for because of a hidden agenda. Whether this is true, and if so, what that agenda may be, beyond specific enhanced cognitive and sensory benefits, remains to be explicated (For a further exploration of the remaining scientific questions, please see Box 1). The picture is complicated not only by the fact that there appear to be many degrees and many variants of the phenomenon (as there is in dyslexia, autism, etc.), but also by the extraordinary phenomenological experiences of some synesthetes (e.g., “5 is not only red but has a grainy texture to it”). But it is precisely because synesthesia seems to occupy that mysterious boundary zone between elementary sensations on the one hand and higher level abstractions (such as gender and personality, and even emotions; e.g., sandpaper evoking the sensation of jealousy [41]) on the other that the phenomenon intrigues us and provides an experimental lever for investigating high-level mental processes. Indeed, Ramachandran and Hubbard [15] pointed out that number personifications in synesthesia (e.g., 5 is male, 7 is female, etc.) may reflect an amplification of the universal human propensity to “binarize” the world to economize cognitive processing.

**Box 1. Remaining Questions**

1. Beyond the vague assertion that synesthesia might enhance sensory and intersensory processing (whether accidentally or “deliberately” selected for), can synesthesia actually enhance sophisticated and abstract mental abilities? Ramachandran and Hubbard [42] pointed out that many synesthetes with visual-spatial number forms [2] claimed such enhancement did occur. When a number-form synesthete imagines or visualizes a number in front of him he always sees it occupying a specific location in space; the numbers are arranged sequentially along a number line that can be highly convoluted in three dimensions—sometimes even doubling back on itself. Many of them said the “kinks” in the line often interfered with their ability to do simple arithmetic across the kinks. Intriguingly some of them reported being able to “see hidden relationships” from unusual vantage points geometrically as a result of the numerical landscape, an effect which could—and should—be measured quantitatively because of the insight it may provide into mathematical computation in normal people as well as savants [15]; indeed, Einstein often remarked that even his “mathematical” thinking was spatial in nature. Finally, we have noted that some number-color synesthetes report difficulty with simple arithmetic because they found the colors “distracting”—an effect we are currently investigating.
2. Synesthesia tends to be a unidirectional phenomenon such that numbers evoke colors but colors won't typically evoke the concept of numbers [15]. This prospect of unidirectionality should constrain theories on the types of anatomical and functional connections that mitigate synesthetic experiences. Presenting a particularly interesting test for the cross-activation theory, research suggests that synesthesia may be unconsciously bidirectional [43]. Future studies investigating the types of anatomical and/or functional connections that mitigate synesthesia will hopefully clarify this matter.
3. What is the relationship between inherited synesthesias (e.g., grapheme-color synesthesia) and acquired conditions and phenomena that produce similar experiences? Specifically, what aspects of synesthesia are based on pharmacology (intensity of the experience, number of different forms of synesthesia within a single individual, or simply possessing synesthesia in general), and how does altering these neurotransmitters affect the experience of synesthesia in the normal population as well as in synesthetes; i.e., does a synesthete who ingests LSD experience novel forms of the condition and/or an enhancement of their current synesthesia? Does a non-synesthete who takes LSD multiple times experience any consistency in these temporary synesthetic associations (e.g., does a 2,000-hz tone elicit a consistent color across multiple sessions)? While the ethical implications of such studies today impede current testing, research from the mid-20th century has provided preliminary evidence relating inherited synesthesia to pharmacologically induced forms: Simpson and McKellar [44] reported on two developmental synesthetes who, under the influence of mescaline, experienced increased vividness of their “natural” synesthetic experiences and also new forms of the phenomenon as well.
4. Do the genes that predispose one to synesthesia independently produce sensory or cognitive enhancements? The specific types of the condition examined in this paper suggest that synesthesia enhances sensory processing. The claim that part of the reason why the phenomenon has survived evolution would be supported if the same benefits are found in all forms of synesthesia. Further, one of the critical links missing from this picture is the question of whether the synesthetic phenotype actually causes these generalized sensory enhancements. This problem can be partially addressed by testing whether family members of synesthetes who themselves are not synesthetic (i.e., carriers) show similar perceptual benefits due to endophenotypes of the synesthesia gene. Additionally, do these individuals show latent synesthetic associations or an enhanced ability to make these associations? No research has been published in this area to date, but our group is currently testing these ideas.
5. Does synesthesia exist in animals? If synesthesia is a phylogenetically old trait it may discount the notion that synesthesia evolved to aid creativity and metaphor in the population. Indeed, the criteria required to successfully create an animal model of synesthesia can be debated as synesthesia is defined by the conscious experience of sensation. Nevertheless, mice with a mutation on α2δ3 show reduced pain sensitivity due to the failure of transmission from the thalamus to the cortex, yet pain instead causes activation of visual and auditory regions similar to the cross-activation seen in synesthesia [45]. It remains to be seen whether this gene is related to synesthesia in humans, but it provides an interesting candidate for research.
6. The phenomena of number-form, weekday-form (each day occupies a specific location in space), and month-form (each month occupies a location in space) synesthesias seem unrelated, yet some synesthetes display all three and in many these sequences evoke colors as well. It remains unclear whether these variants are due to interactions between spatial maps in the parietal lobes and ordinality mechanisms (ordered sequences such as numbers, months, etc.) thought to originate in the vicinity of the angular gyrus as suggested by Ramachandran and Hubbard [15]. Astonishingly, in some instances these spatial-forms change predictably over time; e.g., in month-form synesthesia the calendar will change shape and position to keep the current month in a static position relative to the subject [46]. There is nothing in synesthesia research that can explain such oddities. They remind us both of the fact that we have barely scratched the surface and that synesthesia might give us insights into basic sensory codes used by the brain to represent time and space, topics of which a great deal has been written but nothing is known.
7. If synesthesia involves cross-domain interactions between organized maps of color in V4 and the elementary components of visual forms, and if the disposition of such maps is not random, is there a correlation between the overall shape of the grapheme and color (e.g., do curvy shapes tend to be red and jagged shapes blue)? We have observed that within an individual synesthete correlations exist between shape similarity and color similarity [47], but the question remains whether the layout of hue and shape maps across all individuals predispose particular shape-color correspondences in general. Analogously, are there phoneme maps (depending on where the tongue hits the palette: aspirations, labials, etc.) in cortical motor areas that are non-randomly correlated with evoked colors? This can be partly be tested using an artificially synthesized language like Korean in which the alphabet was constructed based on the position of the tongue on the palette; do letters corresponding to similar tongue positions evoke more similar colors?

In summary, synesthesia is a highly heritable phenomenon that is associated with numerous benefits to cognitive processing, potentially underscoring a basis for why this condition has survived evolutionary pressures. Research into synesthesia is now passing its bicentennial anniversary in science, and understanding both the mechanisms underlying the phenomenon and the reasons for its selection are finally at a point in which synesthesia can inform our understanding of cognitive and perceptual processes in the general population. To appropriately understand this condition and its relation to normal cognition will require both technically and intellectually diverse contributions from all areas of biology. In sum, this research suggests that synesthesia, far from being a “fringe” phenomenon as formerly believed (or that it is purely “conceptual” or associative in nature), can give us vital clues toward understanding some of the physiological mechanisms underlying some of the most elusive yet cherished aspects of the human mind.
